# Association of increased serum glycated albumin levels with low coronary collateralization in type 2 diabetic patients with stable angina and chronic total occlusion

**DOI:** 10.1186/1475-2840-12-165

**Published:** 2013-11-08

**Authors:** Ying Shen, Lin Lu, Feng Hua Ding, Zhen Sun, Rui Yan Zhang, Qi Zhang, Zheng Kun Yang, Jian Hu, Qiu Jing Chen, Wei Feng Shen

**Affiliations:** 1 Department of Cardiology, Shanghai Rui Jin Hospital, Shanghai Jiaotong University School of Medicine, Shanghai 200025, People’s Republic of China; 2 Institute of Cardiovascular Diseases, Shanghai Jiaotong University School of Medicine, Shanghai 200025, People’s Republic of China

**Keywords:** Glycated albumin, Coronary collateralization, Diabetes

## Abstract

**Background:**

We investigated whether serum glycated albumin (GA) levels are related to coronary collateralization in type 2 diabetic patients with chronic total occlusion.

**Methods:**

Blood levels of GA and glycosylated hemoglobin (HbA1c) were determined in 317 diabetic and 117 non-diabetic patients with stable angina and angiographic total occlusion of at least one major coronary artery. The degree of collaterals supplying the distal aspect of a total occlusion from the contra-lateral vessel was graded as low (Rentrop score of 0 or 1) or high collateralization (Rentrop score of 2 or 3).

**Results:**

For diabetic patients, GA (21.2 ± 6.5% *vs.* 18.7 ± 5.6%, *P* < 0.001) but not HbA1c levels (7.0 ± 1.1% *vs.* 6.8 ± 1.3%, *P* = 0.27) was significantly elevated in low collateralization than in high collateralization group, and correlated inversely with Rentrop score (Spearmen’s *r* = -0.28, *P* < 0.001; Spearmen’s *r* = -0.10, *P* = 0.09, respectively). There was a trend towards a larger area under the curve of GA compared with that of HbA1c for detecting the presence of low collateralization (0.64 *vs.* 0.58, *P* = 0.15). In non-diabetic patients, both GA and HbA1c levels did not significantly differ regardless the status of coronary collateralization. In multivariable analysis, female gender, age > 65 years, smoke, non-hypertension, duration of diabetes > 10 years, metabolic syndrome, eGFR < 90 ml/min/1.73 m^2^, and GA > 18.3% were independently determinants for low collateralization in diabetic patients.

**Conclusions:**

Increased GA levels in serum are associated with impaired collateral growth in type 2 diabetic patients with stable angina and chronic total occlusion.

## Background

Coronary collateralization is a physiological adaptive response to transient or permanent occlusion of major coronary arteries [1,2]. Well-developed coronary collaterals contribute to a reduction of infarct size, preservation of left ventricular function, and an improvement of survival in patients with coronary artery disease [3-5]. However, such a physiological response is significantly impaired in patients with type 2 diabetes, metabolic syndrome, and severe atherosclerosis [6,7]. In a diabetic setting, an excessive formation of advanced glycation endproducts (AGEs) and reactive oxygen species (ROS) frequently causes a reduction of endothelium-mediated dilation, while blockade of AGEs restores ischemia-induced angiogenesis [8,9].

Glycated albumin (GA) - a predominant early Amadori-type glycation protein in serum - serves as an alternative measure of dysglycemia over approximately 2–3 weeks, and is associated with the occurrence and severity of atherosclerosis in diabetes [10-14]. In cellular experiment, GA promotes inflammation via activation of NF-κB pathway in endothelial cells, induces adhesion of monocytes to endothelial cells through enhanced transcription of the cell surface adhesion molecules, and stimulates vascular smooth muscle cell proliferation [10]. It is also a prognostic marker for diabetic patients undergoing coronary artery stent implantation [14,15], and a risk factor of mortality and morbidity for those receiving hemodialysis [16,17]. GA could be also formed in a non-diabetic milieu triggered by inflammatory reaction [7,10]. Based upon these findings, it is reasonable to hypothesize that GA might exert a deleterious effect on coronary collateralization in diabetic patients. To verify this hypothesis, we analyzed serum levels of GA in diabetic and non-diabetic patients with stable angina and chronic coronary total occlusion. This angiographic criterion of inclusion was used because a severe coronary artery obstruction was a prerequisite for spontaneous collateral recruitment [18]. We assessed the degree of coronary collateralization according to the Rentrop grading system [19] as this method is easy to incorporate into the routine clinical practice. To the best of our knowledge, there is no previous report referring to the relation of Amadori-modified proteins and collateral formation in such a unique cohort.

## Methods

### Study population

A total of 537 consecutive patients with stable angina and chronic total occlusion (> 3 months) of at least one major epicardial coronary artery between January 2009 and July 2013 from the database of Shanghai Rui Jin Hospital Percutaneous Coronary Intervention (PCI) Outcomes Program were screened. Stable angina was diagnosed according to the criteria recommended by the American College of Cardiology/ American Heart Association [20]. The duration of coronary artery occlusion was estimated from the date of occurrence of myocardial infarction in the area of myocardium supplied by the occluded vessel, from an abrupt worsening of existing angina pectoris, or from information obtained from a previous angiogram. The diagnosis of type 2 diabetes mellitus and dyslipidemia were made according to the criteria of the American Diabetes Association and Third Report of The National Cholesterol Education Program (NCEP) [21,22]. Metabolic syndrome was defined by the updated 2005 National Cholesterol Education Program Adult Treatment Panel III (NCEP ATP III) [23]. We used body mass index (BMI) ≥ 25 kg/m^2^ as the cut point of obesity according to the criteria of Asia-Oceania [24]. For the purpose of research, patients who received PCI within the prior 3 months (n = 41) or had a history of coronary artery bypass grafting (n = 38) were excluded. We also excluded those with chronic heart failure, pulmonary heart disease, malignant tumor or immune system disorders (n = 19). Patients with type 1 diabetes were excluded by measurement of peptide C level (n = 5). The remaining 434 patients (317 diabetics and 117 non-diabetics) were enrolled in this cross-sectional study.

The protocol was approved by the Hospital Ethics Committee and written informed consent was obtained from all patients. Since the study did not involve any intervention, it was not prospectively registered.

### Coronary angiography

Coronary angiography was performed through the femoral or radial approach. All angiograms were reviewed by two experienced interventional cardiologists, according to lesion classification scheme of the American College of Cardiology/American Heart Association [25]. They were blinded to study protocol and biochemical measurements, and any difference in interpretation was resolved by a third reviewer. The presence and degree of collaterals supplying the distal aspect of a total coronary occlusion from the contra-lateral vessel were graded on a 4-point scale from 0 to 3 according to the Rentrop scoring system [18]: zero = no collateral vessels; 1 = thread-like, poorly opacified collaterals with faint visualization of the distal vessel; 2 = moderately opacified collateral channels; 3 = large, brightly filled collateral channels with immediate visualization of the entire distal vessel > 10 mm. Patients were then classified as low (Rentrop score of 0 and 1) and high (Rentrop score of 2 and 3) coronary collateralization, as in previous studies [26,27]. For those with more than one total coronary occlusion, the vessel with the highest collateral grade was chosen for analysis.

### Biochemical measurement

Blood samples were collected at the day of angiography in patients after an overnight fasting. All samples were stored at -80 °C until analysis. Serum glucose, glycosylated hemoglobin (HbA1c), blood urea nitrogen, creatinine, uric acid, and lipid profiles were measured with standard laboratory techniques on a Hitachi 912 Analyzer (Roche Diagnostics, Germany). Modified estimated glomerular filtration rate (eGFR) was calculated, as described previously [28].

Serum GA levels were determined with an improved bromocresolpurple method using Lucica™ glycated albumin-L assay kit (Asahi Kasei Pharma, Japan). The linear range for this assay was 3.2-68.1% and the maximum inter-assay coefficient of variation (CV) was < 3.0% [11,12,14].

### Statistical analysis

Continuous variables are presented as mean and standard deviation (SD), and categorical data are summarized as frequencies or percentages. For categorical clinical variables, differences between groups were evaluated with the chi-square test followed by Bonferroni’s correction to account for multiple comparisons. For continuous variables, the existence of a normal distribution was evaluated with the Kolmolgorov–Smirnov test, and logarithmic or square-root transformations were performed on the continuous variables of non-normal distribution. Differences among groups were analyzed by one-way analysis of variance (ANOVA) or the Kruskal–Wallis analysis followed by post-hoc analysis. Correlation between variables was determined by the Spearman’s rho tests as appropriate. Receiver operating characteristic (ROC) curve was plotted to assess the power of GA and HbA1c for detecting low collateralization, and the area under the curve was compared using the DeLong method. The independent determinants for low collateralization were assessed by multivariate logistic regression analysis, and the covariates chosen to enter the multivariate analysis model included age, gender, BMI, risk factors for coronary artery disease, duration of diabetes, metabolic syndrome, renal impairment, multivessel disease, HbA1c, and GA measurements. All analyses used 2-sided tests with an overall significance level of alpha = 0.05, and were performed with the SPSS 15.0 for Windows (SPSS, Inc., Chicago, IL, USA).

## Results

### Baseline characteristics and bio-measurements

Baseline demographic and clinical characteristics and biochemical measurements are listed in Table 1. Both diabetic and non-diabetic patients with low collateralization were older and less hypertensive, but were females in higher percentage and had higher fasting glucose and more dyslipidemia, and renal impairment than those with high collateralization (for all comparison, *P* < 0.05). Metabolic syndrome was more common, and serum levels of total cholesterol, low-density lipoprotein cholesterol, and uric acid were significantly elevated in diabetic patients with low collateralization.

**Table 1:** Baseline characteristics and biochemical assessment in patients with chronic total occlusion

		**Non-diabetes**			**Diabetes**	
**Collateralization**	**Low**	**High**	**P value**	**Low**	**High**	**P value**
Number of patients	n = 22	n = 95		n = 118	n = 199	
Female, n (%)	12 (54.5)	12 (12.6)	< 0.001	41 (34.7)	27 (13.6)	< 0.001
Age, y	68 ± 11.1	62.4 ± 10.4	0.03	66.5 ± 10.1	64.6 ± 11.1	0.12
Age > 65y	16 (72.7)	33 (34.7)	0.001	76 (64.4)	80 (40.2)	< 0.001
Body mass index, Kg/m^2^	25.3 ± 2.8	24.8 ± 3.0	0.53	25.8 ± 3.4	25.1 ± 3.4	0.07
Smoke, n (%)	8 (36.4)	47 (49.5)	0.27	46 (39.0)	71 (35.7)	0.56
Hypertension, n (%)	10 (45.5)	69 (72.6)	0.01	77 (65.3)	157 (78.9)	0.008
Dyslipidemia, n (%)	15 (68.2)	37 (38.9)	0.01	67 (56.8)	81 (40.7)	0.006
Systolic blood pressure, mm Hg	142.3 ± 18.3	139.6 ± 17.0	0.51	140.5 ± 18.7	139.9 ± 17.1	0.76
Diastolic blood pressure, mm Hg	86.4 ± 13.6	84.4 ± 8.1	0.53	84.1 ± 10.4	84.3 ± 9.1	0.86
Durration of diabetes, y	/	/	/	8.8 ± 4.6	5.7 ± 3.4	< 0.001
Fasting blood glucose, mmol/L	5.4 ± 0.7	5.0 ± 0.7	0.03	5.9 ± 1.8	5.5 ± 1.5	0.049
HbA1c,%	6.3 ± 0.3	6.3 ± 0.5	0.86	7.0 ± 1.1	6.8 ± 1.3	0.27
Glycated albumin,%	19.1 ± 4.6	18.4 ± 5.2	0.54	21.2 ± 6.5	18.7 ± 5.6	< 0.001
Triglyceride, mmol/L	1.8 ± 0.8	1.7 ± 0.8	0.49	1.9 ± 1.1	1.8 ± 1.2	0.36
Total cholesterol, mmol/L	3.9 ± 1.1	4.4 ± 1.1	0.08	4.5 ± 1.5	4.1 ± 1.2	0.009
HDL-C, mmol/L	0.9 ± 0.2	1.0 ± 0.3	0.10	1.0 ± 0.3	1.1 ± 0.3	0.10
LDL-C, mmol/L	2.4 ± 1.0	2.7 ± 0.9	0.15	2.8 ± 1.2	2.5 ± 1.0	0.045
Metabolic syndrome, n (%)	12 (54.5)	42 (44.2)	0.38	106 (89.8)	148 (74.4)	0.001
Blood urea nitrogen, mmol/L	5.1 ± 1.7	5.0 ± 2.0	0.82	5.3 ± 2.3	5.1 ± 1.6	0.40
Uric acid, mmol/L	354.5 ± 59.4	337.0 ± 83.7	0.36	353.3 ± 84.6	329.7 ± 77.4	0.01
Creatinine, μmol/L	85.2 ± 17.1	76.4 ± 22.3	0.08	89.7 ± 22.5	76.8 ± 23.7	< 0.001
eGFR, mL/min/1.73 m^2^	80.9 ± 24.3	104.3 ± 26.3	< 0.001	80.5 ± 21.5	102.4 ± 24.1	< 0.001
Number of significant CAD			0.25			0.30
1-vessel	8 (36.4)	19 (20.0)		30 (25.4)	38 (19.1)	
2-vessel	9 (40.9)	46 (48.4)		40 (33.9)	82 (41.2)	
3-vessel	5 (22.7)	30 (31.6)		48 (40.7)	79 (39.7)	
Medication, n (%)						
ACE inhibitors	7 (31.8)	33 (34.7)	0.80	63 (53.4)	106 (53.3)	0.98
β-blockers	11 (50.0)	28 (29.5)	0.07	44 (37.3)	78 (39.2)	0.74
Calcium channel blockers	7 (31.8)	20 (21.1)	0.28	32 (27.1)	51 (25.6)	0.77
Nitrates	5 (22.7)	35 (36.8)	0.21	69 (58.5)	127 (63.8)	0.34
Statins^*^	18 (81.2)	82 (86.3)	0.89	98 (83.7)	166 (83.3)	0.99
Anti-diabetic treatment	0 (0)	0 (0)	/	92 (78.0)	170 (85.4)	0.09

Data are mean ± SD or number (%). *Abbreviations:* ACE, angiotensin-converting enzyme; eGFR, estimated glomerular filtration rate; HbA1c, glycatedhemoglobin A1c; HDL-C, high-density lipoprotein cholesterol; LDL-C, low- density lipoprotein cholesterol *Statins: mainly simvastatin, pravastatin and atorvastatin.

### Serum GA levels and coronary collateralization

**Figure 1:**
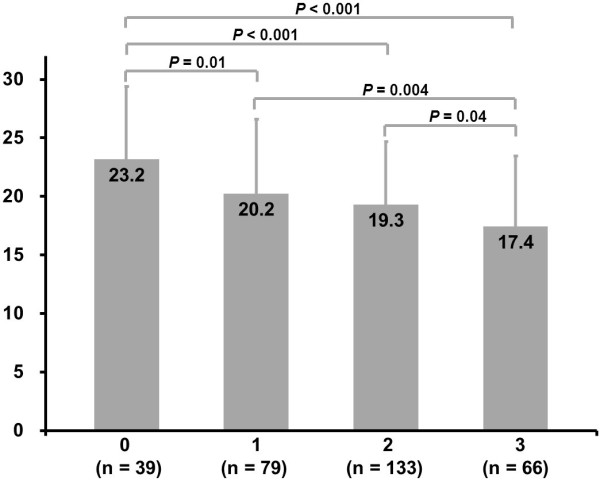
Relationship between serum levels of glycated albumin and collateral flow grade in diabetic patients.

GA levels (21.2 ± 6.5% *vs.* 18.7 ± 5.6%, *P* < 0.001) but not HbA1c (7.0 ± 1.1% *vs.* 6.8 ± 1.3%, *P* = 0.27) were consistently higher in diabetic patients with low collateralization as compared with those who had high collateralization (Table 1). This finding was persistent even after having the group age-matched (*P* = 0.004 and *P* = 0.11, respectively). Further statistics showed that subgroups stratified by Rentrop score exhibited significant difference in GA levels for diabetic patients (Figure 1), and GA but not HbA1c correlated inversely with the degree of collateralization categorized by Rentrop score after adjusting for age, gender, BMI, risk factors for coronary artery disease (including smoke, hypertension and dyslipidemia), multivessel disease, and eGFR (Spearmen’s *r* = -0.28, *P* < 0.001; Spearmen’s *r* = -0.10, *P* = 0.09, respectively). In contrast, for non-diabetic counterparts, both GA and HbA1c levels did not significantly differ regardless the status of coronary collateralization and after having the group age-matched (*P* = 0.96 and *P* = 0.80, respectively).

**Figure 2:**
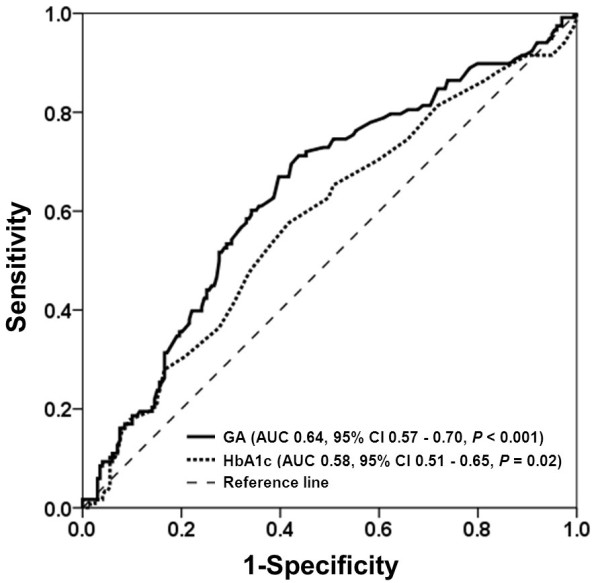
ROC curve for glycated albumin (GA) and glycated hemoglobin (HbA1c) in detecting the presence of low collateralization in diabetic patients.

ROC curve analysis showed that there was a trend towards a larger area under the curve of GA compared with that of HbA1c (0.64 *vs.* 0.58, *P* = 0.15), and the cut-off of GA > 18.3% was more sensitive than HbA1c > 7.0% for detecting the presence of low collateralization in diabetic patients (67% *vs.* 47%, Figure 2).

### Multivariable analysis

**Figure 3:**
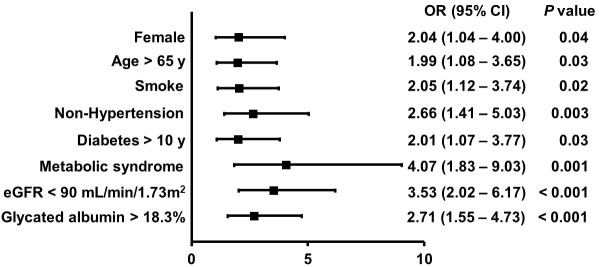
**Relative risk for low collateralization in diabetic patients.** Data are ORs, with 95% CI in parentheses. (eGFR, estimated glomerular filtration rate).

Multivariate logistic regression analysis revealed that in non-diabetic patients, female gender (*P* = 0.01), age > 65 years (*P* = 0.04), non-hypertension (*P* = 0.02), dyslipidemia (*P* = 0.02), and eGFR < 90 mL/min/1.73 m^2^ (*P* = 0.04) were independently associated with low collateralization. In diabetic patients, after adjusting for possible confounding factors including age, gender, BMI, risk factors for coronary artery disease, duration of diabetes, metabolic syndrome, impaired renal function, and multivessel disease, GA > 18.3% but not HbA1c > 7% remained an independent determinant for low collateralization (Figure 3).

## Discussion

Coronary collateral circulation is an important prognostic marker of ischemic heart disease [5]. This study is the first to demonstrate that increased serum GA levels are inversely associated with the degree of coronary collateral formation in diabetic patients with stable angina and chronic total occlusion, adding novel information on pathophysiology of impaired collateral growth in diabetes.

Accumulating evidence suggests that diabetes may exert a deleterious effect on several components necessary for collateral development, including pro-angiogenic growth factors, endothelial function, redox state of the coronary circulation, intracellular signaling, leukocytes and bone marrow-derived progenitor cells [6]. Previous studies have shown that GA induces endothelial dysfunction, and produces pro-inflammatory effects in macrophages through ROS augmentation [6,29-32]. Moreover, this glycated protein reflecting poor glycemic control impairs angiogenic function and elicits apoptosis of endothelial progenitor cells (EPCs) [33-37]. Thus, these observations support a notion that GA may be either a risk factor or a marker for poor collateral growth in diabetes.

In the present study, at least mildly impaired renal function (eGFR < 90 ml/min/1.73 m^2^) was found to be a strong independent risk factor for low collateralization in patients with stable angina and chronic total occlusion, particularly in those with diabetes. This finding may be partly related to a high occurrence rate of chronic kidney disease in diabetic and non-diabetic patients with coronary artery disease, which is supported by prior reports [26,38,39]. The direct and indirect effects of uremic toxins cause ROS-mediated endothelial dysfunction and inhibit proliferation of EPCs [40], combined with pathophysiology of diabetes including the effect of GA, eventually lead to reduced collateral growth in patients with chronic kidney disease.

Notably, the present study showed that GA but not HbA1c levels were higher in diabetic patients with low coronary collateralization, and that GA levels were more sensitive for detecting low coronary collateralization as compared with HbA1c in diabetic patients. These results are consistent with previous observations including ours, indicating that GA is superior to HbA1c in evaluating vasculopathies in diabetic patients with certain specific disorders such as coronary artery disease and renal dysfunction [41].

### Limitations

We recognize that there are several limitations in our study. First, the study is cross-sectional for the point of coronary collateral investigation, thereby allowing us to detect association, not to predict outcome. Second, we evaluated the presence and degree of collaterals according to the Rentrop scoring system. Coronary collaterals may be more accurately assessed by collateral flow index with simultaneous measurement of aortic pressure and the distal pressure within the occluded segment of the culprit coronary artery [1]. Nevertheless, angiographic assessment of coronary collaterals is easy to incorporate into the routine clinical practice [19,26]. Third, elevated serum GA levels and low coronary collateralization may be also caused by the presence of metabolic syndrome and not diabetes alone. Finally, further large-scale studies with molecular experiments are required to clarify the mechanisms of GA effect on coronary collateralization.

## Conclusions

The present study has demonstrated an association between increased GA levels in serum and reduced coronary collaterals in diabetic patients with stable angina and chronic total occlusion. These findings might offer a rationale in an effort to improve clinical outcomes in this unique diabetic cohort with aggressive intervention of AGEs and inflammatory process.

### Source of funding

This study was supported by the National Natural Science Foundation of China (81070240 and 81070178) and Science Technology Committee of Shanghai Municipal Government (10JC1410500 and 2011019).

## Abbreviations

AGEs: Advanced glycation endproducts; ANOVA: Analysis of variance; CV: Coefficient of variation; eGFR: estimated glomerular filtration rate; EPCs: Endothelial progenitor cells; GA: Glycated albumin; HbA1c: Glycosylated hemoglobin; PCI: Percutaneous coronary intervention; ROC: Receiver operating characteristic; ROS: Reactive oxygen species; SD: Standard deviation.

## Competing interests

The authors declare that they have no competing interests.

## Authors’ contributions

YS performed the study. WFS designed the studies and drafted the manuscript. LL and ZS collected the data. FHD did statistical analysis. RYZ, QZ, ZKY, JH performed angiography and imaging assessment. QJC made biochemical analysis. All authors have read and approved the final manuscript.

## References

[B1] Traupe T Gloekler S de Marchi SF Werner GS Seiler C Assessment of the human coronary collateral circulation Circulation 2010 122 12 1210 1220 10.1161/CIRCULATIONAHA.109.930651 20855668

[B2] Schaper W Collateral circulation, past and present Basic Res Cardiol 2009 104 1 5 21 10.1007/s00395-008-0760-x 19101749 PMC2755790

[B3] Habib GB Heibig J Forman SA Brown BG Roberts R Terrin ML Bolli R the TIMI investigators: Influence of coronary collateral vessels on myocardial infarction size in humans: results of phase I Thrombolysis in Myocardial Infarction (TIMI) trial Circulation 1991 83 3 739 746 10.1161/01.CIR.83.3.739 1900223

[B4] Meier P Gloekler S Zbinden R Beckh S de Marchi SF Zbinden S Wustmann K Billinger M Vogel R Cook S Beneficial effects of recruitable collaterals, a 10-year follow-up study in patients with stable coronary artery disease undergoing quantitative collateral measurements Circulation 2007 116 9 975 983 10.1161/CIRCULATIONAHA.107.703959 17679611

[B5] Meier P Hemingway H Lansky AJ Knapp G Pitt B Seiler C The impact of the collateral circulation on mortality: a meta-analysis Eur Heart J 2012 33 5 614 621 10.1093/eurheartj/ehr308 21969521

[B6] Rocic P Why is coronary collateral growth impaired in type II diabetes and the metabolic syndrome? Vascul Pharmacol 2012 57 5–6 179 186 22342811 10.1016/j.vph.2012.02.001 PMC3359422

[B7] Turgut O Yilmaz MB Yalta K Tandogan I Yilmaz A Prognostic relevance of coronary collateral circulation: clinical and epidemiological implications Int J Cardiol 2009 137 3 300 301 10.1016/j.ijcard.2008.06.016 18684526

[B8] Vessieres E Freidja ML Loufrani L Fassot C Henrion D Flow (shear stress)-mediated remodeling of resistance arteries in diabetes Vascul Parmacol 2012 57 5–6 173 178 10.1016/j.vph.2012.03.006 22484164

[B9] Tamarat R Silvestre JS Huijberts M Benessiano J Ebrahimian TG Duriez M Wautier MP Wautier JL Levy BI Blockade of advanced glycation end-product formation restores ischemia-induced angiogenesis in diabetic mice Proc Natl Acad Sci U S A 2003 100 14 8555 8560 10.1073/pnas.1236929100 12805564 PMC166267

[B10] Cohen MP Ziyadeh FN Chen S Amadori-modified glycated serum proteins and accelerated atherosclerosis in diabetes: pathogenic and therapeutic implications J Lab Clin Med 2006 147 5 211 219 10.1016/j.lab.2005.12.006 16697768 PMC1800931

[B11] Pu LJ Lu L Xu XW Zhang RY Zhang Q Zhang JS Hu J Yang ZK Ding FH Chen QJ Value of serum glycated albumin and high-sensitivity C-reactive protein levels in the prediction of presence of coronary artery disease in patients with type 2 diabetes Cardiovasc Diabetol 2006 5 27 10.1186/1475-2840-5-27 17178005 PMC1764721

[B12] Lu L Pu LJ Zhang Q Wang LJ Kang S Zhang RY Chen QJ Wang JG De Caterina R Shen WF Increased glycated albumin and decreased esRAGE levels are related to angiographic severity and extent of coronary artery disease in patients with type 2 diabetes Atherosclerosis 2009 206 2 540 545 10.1016/j.atherosclerosis.2008.12.045 19368923

[B13] Furusyo N Koga T Ai M Otokozawa S Kohzuma T Ikezaki H Schaefer EJ Hayashi J Plasma glycated albumin level and atherosclerosis: results from the Kyushu and Okinawa Population Study (KOPS) Int J Cardiol 2013 167 5 2066 2072 10.1016/j.ijcard.2012.05.045 22658569

[B14] Shen Y Pu LJ Lu L Zhang Q Zhang RY Shen WF Glycated albumin is superior to hemoglobin A1c for evaluating the presence and severity of coronary artery disease in type 2 diabetic patients Cardiology 2012 123 2 84 90 10.1159/000342055 23018602

[B15] Shen Y Pu LJ Lu L Zhang Q Zhang RY Shen WF Serum advanced glycation endproducts and receptors as prognostic biomarkers in diabetic undergoing coronary artery stent implantation Can J Cardiol 2012 28 6 737 743 10.1016/j.cjca.2012.08.015 23073352

[B16] Biesenbach G Pohanka E Dialysis: glycated albumin or HbA1c in dialysis patients with diabetes? Nature Reviews Nephology 2011 7 9 490 492 10.1038/nrneph.2011.95 21808280

[B17] Shafi T Sozio SM Plantinga LC Jaar BG Kim ET Parekh RS Steffes MW Powe NR Coresh J Selvin E Serum fructosamine and glycated albumin and risk of mortality and clinical outcomes in hemodialysis patients Diabetes Care 2013 36 6 1522 1533 10.2337/dc12-1896 23250799 PMC3661814

[B18] Levin DC Pathways and functional significance of the coronary collateral circulation Circulation 1974 50 4 831 837 10.1161/01.CIR.50.4.831 4425386

[B19] Rentrop KP Cohen M Blanke H Phillips RA Changes in collateral channel filling immediately after controlled coronary artery occlusion by an angioplasty balloon in human subjects J Am Coll Cardiol 1985 5 3 587 592 10.1016/S0735-1097(85)80380-6 3156171

[B20] Fraker TD Jr Fihn SD Gibbons RJ Abrams J Chatterjee K Daley J Deedwania PC Douglas JS Ferguson TB Jr Gardin JM 2007 chronic angina focused update of the ACC/AHA 2002 guidelines for the management of patients with chronic stable angina: a report of the American College of Cardiology/American Heart Association Task Force on Practice Guidelines Writing Group to develop the focused update of the 2002 guidelines for the management of patients with chronic stable angina J Am Coll Cardiol 2007 50 23 2264 2274 10.1016/j.jacc.2007.08.002 18061078

[B21] Expert Committee on the Diagnosis and Classification of Diabetes Mellitus Report of the expert committee on the diagnosis and classification of diabetes mellitus Diabetes Care 2003 26 Suppl 1 S5 S20 12502614 10.2337/diacare.26.2007.s5

[B22] Executive Summary of The Third Report of The National Cholesterol Education Program (NCEP) Expert Panel on Detection, Evaluation, And Treatment of High Blood Cholesterol In Adults (Adult Treatment Panel III) JAMA 2001 285 19 2486 2497 10.1001/jama.285.19.2486 11368702

[B23] Grundy SM Cleeman JI Daniels SR Donato KA Eckel RH Franklin BA Gordon DJ Krauss RM Savage PJ Smith SC Jr Spertus JA Costa F American Heart Association; National Heart, Lung, and Blood Institute. Diagnosis and management of the metabolic syndrome: an American Heart Association/National Heart, Lung, and Blood Institute Scientific Statement Circulation 2005 112 17 2735 2752 10.1161/CIRCULATIONAHA.105.169404 16157765

[B24] Kanazawa M Yoshike N Osaka T Numba Y Zimmet P Inoue S Criteria and classification of obesity in Japan and Asia-Oceania Asia Pac J Clin Nutr 2002 11 suppl 8 s732 s737 16145245 10.1159/000088200

[B25] Ellis SG Vandormael MG Cowley MJ DiSciascio G Deligonul U Topol EJ Bulle TM Coronary morphologic and clinical determinants of procedural outcome with angioplasty for multivessel coronary disease: Implications for patient selection (Multivessel Angioplasty Prognosis Study Group) Circulation 1990 82 4 1193 1202 10.1161/01.CIR.82.4.1193 2401060

[B26] Hsu PC Juo SH Su HM Chen SC Tsai WC Lai WT Sheu SH Lin TH Predictor of poor coronary collaterals in chronic kidney disease population with significant coronary artery disease BMC Nephrol 2012 13 98 10.1186/1471-2369-13-98 22935602 PMC3457843

[B27] Kadi H Ozyurt H Ceyhan K Koc F Celik A Burucu T The relationship between high-density lipoprotein cholesterol and coronary collateral circulation in patients with coronary artery disease J Invest Med 2012 60 5 808 812 10.2310/JIM.0b013e31824e980c 22460232

[B28] Ma YC Zuo L Chen JH Luo Q Yu XQ Li Y Xu JS Huang SM Wang LN Huang W Modified glomerular filtration rate estimating equation for Chinese patients with chronic kidney disease J Am Soc Nephrol 2006 17 10 2937 2944 10.1681/ASN.2006040368 16988059

[B29] Rodino-Janeiro BK Gonzalez-Peteiro M Ucieda-Somoza R Gonzalez-Juanatey JR Alvarez E Glycated albumin, a precursor of advanced glycation end products, up-regulates NADPH oxidase and enhances oxidative stress in human endothelial cells: molecular correlate of diabetic vasculopathy Diabetes Metab Res Rev 2010 26 7 550 558 10.1002/dmrr.1117 20818804

[B30] Rubenstein DA Morton BE Yin W The combined effects of sidestream smoke extracts and glycated albumin on endothelial cells and platelets Cardiovasc Diabetol 2010 9 28 10.1186/1475-2840-9-28 20604957 PMC2909174

[B31] Cohen MP Shea E Chen S Shearman CW Glycated albumin increases oxidase stress, activates NF-κB and extracellular signal-regulated kinase (ERK), and stimulates ERK-dependent transforming growth factor-beta 1 production in macrophage RAW cells J Lab Clin Med 2003 141 4 242 249 10.1067/mlc.2003.27 12677169

[B32] Baraka-Vidot J Guerin-Dubourg A Dubois F Payet B Bourdon E Rondeau P New insights into deleterious impacts of in vivo glycation on albumin antioxidant activities Biochim Biophys Acta 2013 1830 6 3532 3541 10.1016/j.bbagen.2013.01.019 23376313

[B33] Scheubel RJ Kahrstedt S Weber H Holtz J Friedrich I Borgerman J Silber RE Simm A Depression of progenitor cell function by advanced glycation endproducts (AGEs): potential relevance for impaired angiogenesis in advanced age and diabetes Exp Gerontol 2006 41 5 540 548 10.1016/j.exger.2006.01.002 16515851

[B34] Li H Zhang X Guan X Cui X Wang Y Chu H Cheng M Advanced glycation end products impair the migration, adhesion and secretion potentials of late endothelial progenitor cells Cardiovasc Diabetol 2012 11 46 10.1186/1475-2840-11-46 22545734 PMC3403843

[B35] Sun C Liang C Ren Y Zhen Y He Z Wang H Tan H Pan X Wu Z Advanced glycation end products depress function of endothelial progenitor cells via p38 and ERK1/2 mitogen-activated protein kinase pathways Basic Res Cardiol 2009 104 1 42 49 10.1007/s00395-008-0738-8 18622638

[B36] Menegazzo L Albiero M Avogaro A Fadini GP Endothelial progenitor cells in diabetes mellitus Biofactors 2012 38 3 194 202 10.1002/biof.1016 22488933

[B37] Yue WS Lau KK Siu CW Wang M Yan GH Yiu KH Tse HF Impact of glycemic control on circulating endothelial progenitor cells and arterial stiffness in patients with type 2 diabetes mellitus Cardiovasc Diabetol 2011 10 113 10.1186/1475-2840-10-113 22185563 PMC3258289

[B38] Xie SL Li HY Deng BQ Luo NS Geng DF Wang JF Nie RQ Poor coronary collateral vessel development in patients with mild to moderate renal insufficiency Clin Res Cardiol 2011 100 3 227 233 10.1007/s00392-010-0233-8 20865265

[B39] Kadi H Ceyhan K Sogut E Koc F Celik A Onalan O Sahin S Mildly decreased glomerular filtration rate is associated with poor coronary collateral circulation in patients with coronary artery disease Clin Cardiol 2011 34 10 517 621 10.1002/clc.20951 PMC6652497 21887692

[B40] Ying Y Yang K Liu Y Chen QJ Shen WF Lu L Zhang RY A uremic solute, P-cresol, inhibits the proliferation of endothelial progenitor cells via the p38 pathway Circ J 2011 75 9 2252 2259 10.1253/circj.CJ-11-0046 21747198

[B41] Lu L Pu LJ Xu XW Zhang Q Zhang RY Zhang JS Hu J Yang ZK Lu AK Ding FH Shen J Chen QJ Lou S Fang DH Shen WF Association of serum levels of glycated albumin, C-reactive protein and tumor necrosis factor-alpha with the severity of coronary artery disease and renal impairment in patients with type 2 diabetes mellitus Clin Biochem 2007 40 11 810 816 10.1016/j.clinbiochem.2007.03.022 17499233

